# Supplementary material for: Therapeutic correction of ApoER2 splicing in Alzheimer's disease mice using antisense oligonucleotides
Source: EMBO Mol Med. 2016 Feb 22;8(4):328–45. doi: 10.15252/emmm.201505846 (PMC4818756; doi:10.15252/emmm.201505846)
Supplement: Supplementary file 1 — Appendix [file EMMM-8-328-s001.pdf]

Appendix Table S1

Antisense Oligonucleotides, siRNA and Primers

2'MOE ASOs	
18-mers (Mouse)	
#	Sequence (5'-3')
1	CCTGTTCTAACCGCTTCA
2	CGACACCTGTTCTAACCG
3	AACAACGACACCTGTTCT
4	TGTGCAACAACGACACCT
5	TCTGCTGTGCAACAACGA
6	CTGACTCTGCTGTGCAAC
7	GCCCCCTGACTCTGCTGT
8	ACTCAGCCCCCTGACTCT
9	GTCATACTCAGCCCCCTG
10	CTGAGGTCATACTCAGCC
11	GACCTCTGAGGTCATACT
12	TGGATGACCTCTGAGGTC
13	TGGACTGGATGACCTCTG
14	AGAGTGGCACTGCCCTAC
15	GGCTCAGAGTGGCACTGC
16	CCTCAGGCTCAGAGTGGC
17	CAGCCCCTCAGGCTCAGA
18	CTCGCCAGCCCCTCAGGC
19	CCTCACTCGCCAGCCCCT
20	AAGGCCCTCACTCGCCAG
21	GGTTTAAGGCCCTCACTC
22	GCCTAGGTTTAAGGCCCT
23	TAGCTGCCTAGGTTTAAG
24	TTCTATAGCTGCCTAGGT
25	GGATTTTCTATAGCTGCC
26	TGCTAGGATTTTCTATAG
27	AAGCCTGCTAGGATTTTC
28	TCAGAAAGCCTGCTAGGA
control	TTAGTTTAATCACGCTCG
18-mers (Human)	
1	CCTGCTGTGCCACTTTGT
2	TGACACCTGCTGTGCCAC
3	AACAGTGACACCTGCTGT
4	TGTATAACAGTGACACCT
5	TGTGCTGTATAACAGTGA
6	CTGACTGTGCTGTATAAC

7	GCCCCCTGACTGTGCTGT
8	ACTCAGCCCCCTGACTGT
9	GTCACACTCAGCCCCCTG
10	CTGAGGTCACACTCAGCC
11	GACCTCTGAGGTCACACT
12	CACTGTGGATGACCTCTG
13	GACTTAGAGTGGCACTGC
14	CCTCAGACTTAGAGTGGC
15	CAGCCCCTCAGACTTAGA
16	CTCACCAGCCCCTCAGAC
17	CCTCACTCACCAGCCCCT
18	CATGCCCTCACTCACCAG
19	TGGTTCATGCCCTCACTC
20	TGCCTTGGTTCATGCCCT
21	GCGCCTGCCTTGGTTCAT
22	TGGCTGCGCCTGCCTTGG
23	AGGATTGGCTGCGCCTGC
24	CTTCTAGGATTGGCTGCG
25	AAAGCCTTCTAGGATTGG
26	ACCAGAAAGCCTTCTAGG
siRNA	
Mm_Sfrs1_1	Target: UACCAGUAAACUUCAAUUAUA (# SIO1415883, Qiagen)
Hs_Sfrs1_1	5’ GCAGAUGAACUCGGGAUG 3’
Primers	Sequence (5'-3')
APOER2ex18F	TGGTGATAGCCCTCCTGTG
APOER2ex20R	TGCATGGGACTGAATTCC
musAPOERex18F	TGGTAATAGCCCTGCTATG
musAPOERex20R	TGCATAGGACTGAACTCC
TgCRND8_F	TGTCCAAGATGCAGCAGAACGGCTACGAAAA
TgCRND8_R	AGAAATGAAGAAACGCCAAGCGCCGTGACT
