# Supplementary material for: Therapeutic correction of ApoER2 splicing in Alzheimer's disease mice using antisense oligonucleotides
Source: EMBO Mol Med. 2016 Feb 22;8(4):328–45. doi: 10.15252/emmm.201505846 (PMC4818756; doi:10.15252/emmm.201505846)
Supplement: Supplementary file 2 — Expanded View Figures PDF [file EMMM-8-328-s002.pdf]

Expanded View Figures

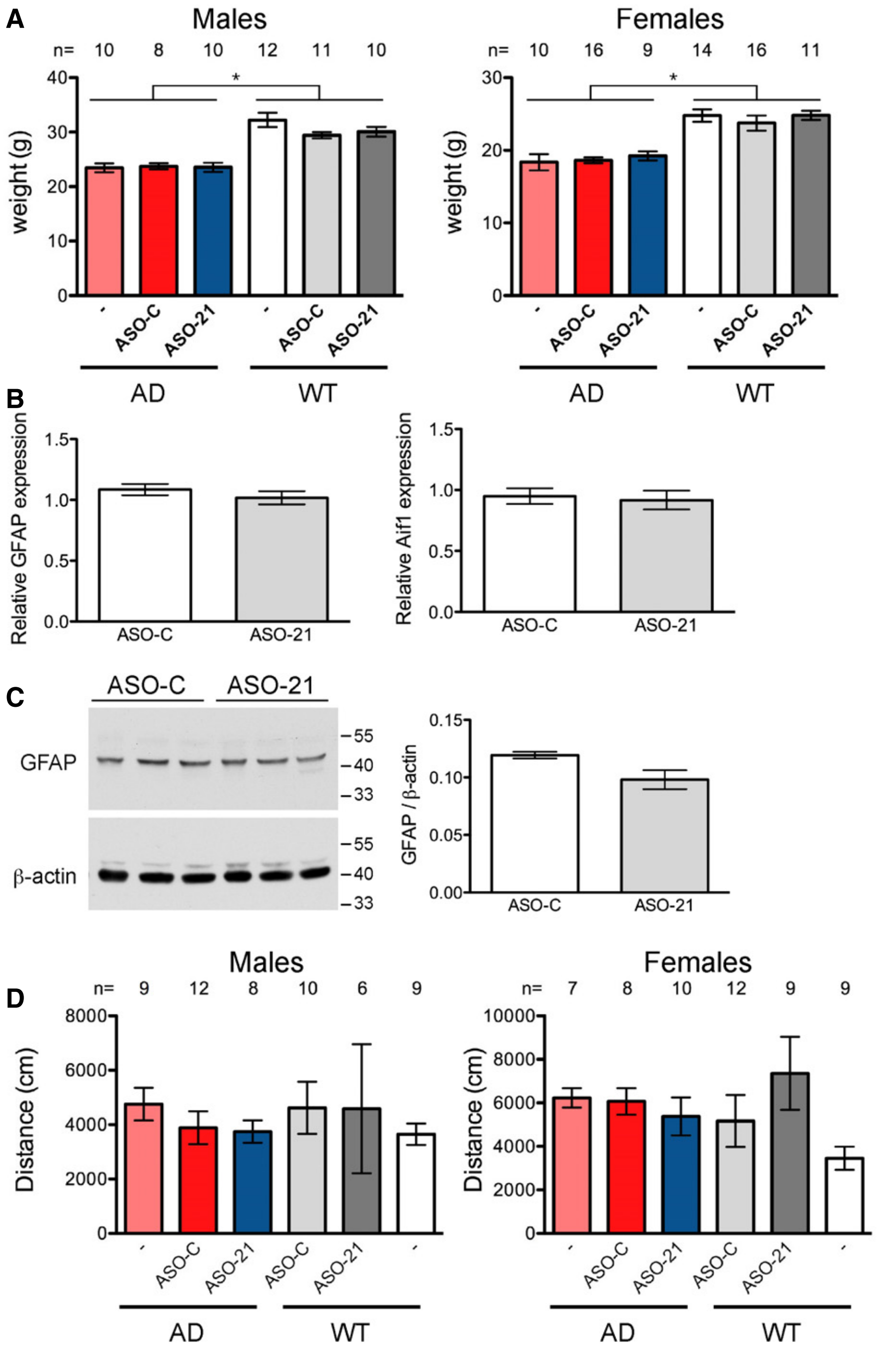

Figure EV1. WT and AD mouse weights and open-field analysis.

- A Weights of 3-month-old WT and AD mice. Weights were not significantly different among the AD groups (males: KW = 0.028, P = 0.986; females: KW = 1.714, P = 0.42) or WT (males: KW = 3.22, P = 0.20; females: KW = 2.53, P = 0.28) groups. AD groups were significantly different than WT groups (males: KW = 44.48, $P < 0.0001$; females: KW = 42.86, $P < 0.0001$). The number of samples analyzed (n) is indicated.
- B Microglia activation and astrocytosis was measured by GFAP and Aif1 qRT–PCR, respectively. RNA for analysis was isolated from the cortex of mice 4 months after ASO treatment. Student's t-test, two-tailed, was performed on the results, n = 3 per group, mean ± s.e.m.
- C Immunoblot analysis of GFAP (astrocyte marker) was also performed to further assess astrocytosis. β-actin was used as a loading control. Quantification of proteins is shown on the right (mean ± s.e.m., $P > 0.05$, Student's t-test).
- D Open-field analysis. The overall activity level of the mice was assessed in an open-field chamber. There was not a significant difference between the groups in the total distance travelled during the 10-min time period of the test (Kruskal–Wallis test, males: KW = 3.72, P = 0.59; females: KW = 10.65, P = 0.06). The number of samples analyzed (n) is indicated.

Source data are available online for this figure.

© 2016 The Authors

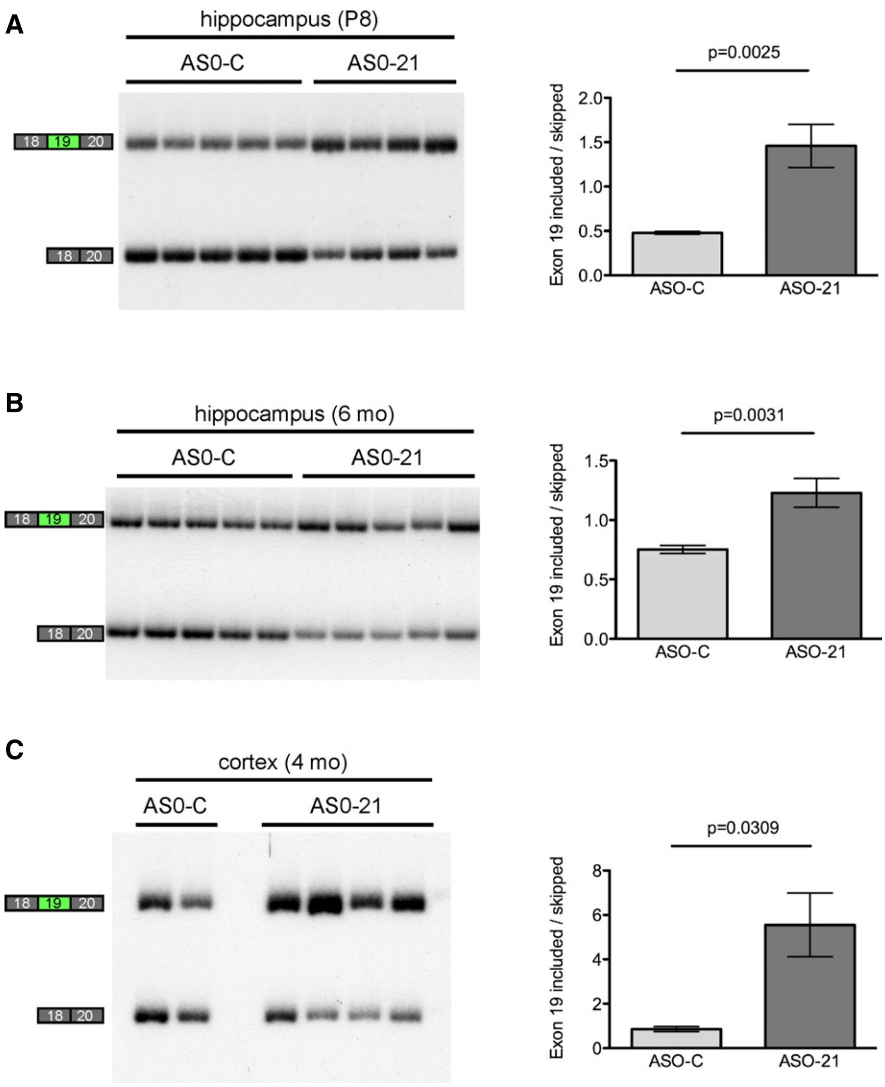

Figure EV2. ASO-21 increases *ApoER2* exon 19 inclusion at 1 week and up to 6 months after treatment.

A RT–PCR analysis of RNA isolated from the hippocampus of P8 TgCRND8 (AD) mice that were treated with ASO-C (n = 5) or ASO-21 (n = 4) by ICV injection at P2. Quantitation is shown to the right of the gel images (mean ± s.e.m., Student's t-test).

B RT–PCR analysis of RNA isolated from the hippocampus of 6-month-old WT and AD mice that were treated with ASO-C (n = 6) or ASO-21 (n = 11) by ICV injection at P1 or P2. Quantitation is shown to the right of the gel images (mean ± s.e.m., Student's t-test).

C RT–PCR analysis of RNA isolated from the cortex of 4-month-old AD mice that were treated with ASO-C (n = 2) or ASO-21 (n = 4) by ICV injection at P1 or P2. Quantitation is shown to the right of the gel images (mean ± s.e.m., Student's t-test).

Source data are available online for this figure.

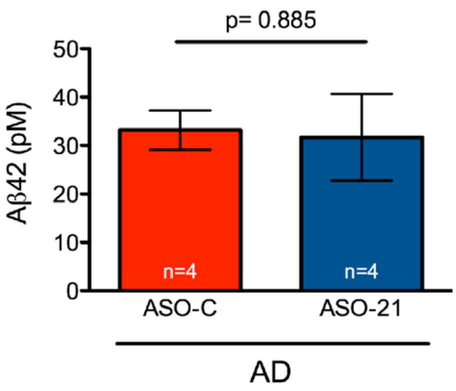

Figure EV3. Aβ peptide abundance is not affected by ASO-21 treatment in AD mice.

Graph shows Aβ42 abundance in the cortex of mice treated with ASO-C or ASO-21 (mean ± s.e.m., Student's t-test) as measured by ELISA. The number of samples analyzed (n) is indicated.

© 2016 The Authors

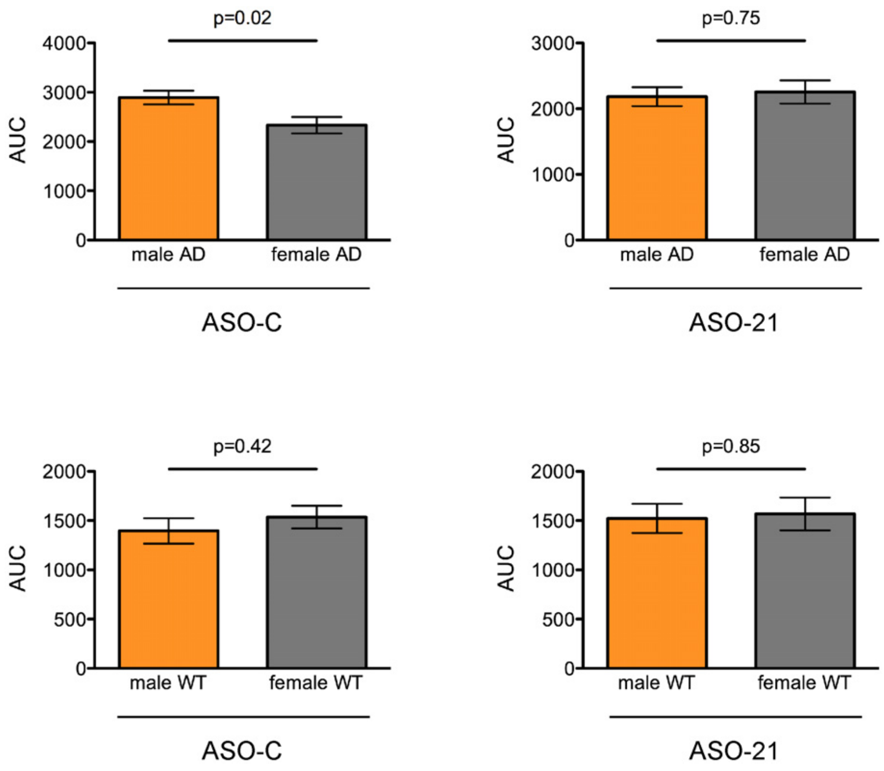

Figure EV4. Area under the curve analysis of Morris water maze data.
Morris water maze analysis integrated distance area under the curve (AUC) for male and female AD (top) and WT (bottom) mice treated with control ASO (left) or ASO-21 (right)(mean ± s.e.m.; Student's *t*-test). Mice are the same as those analyzed in Fig 6.

© 2016 The Authors

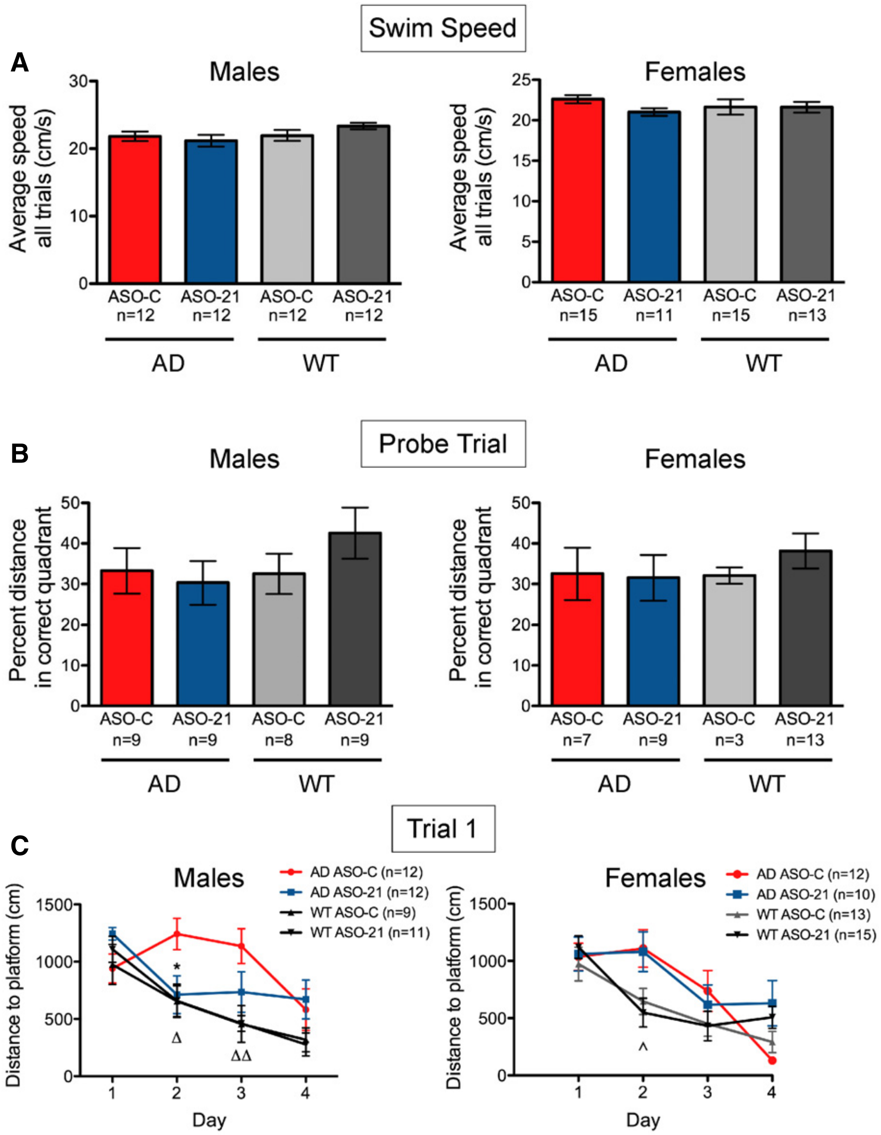

Figure EV5. Morris water maze swim speed and probe trial analysis.

A Quantitation of the swim speed of the mice in the Morris water maze task. The numbers represent the average for each mouse on each of the 4 days of the test (mean ± s.e.m.). The swim speeds were not significantly different among any of the male (Kruskal–Wallis test, KW = 5.77, P = 0.12) or female (Kruskal–Wallis, KW = 7.846, P = 0.05) groups using Dunn's multiple comparison test.

B Probe trial result (mean ± s.e.m.; P = 0.4256 (males), P = 0.7576 (females), one-way ANOVA).

C Morris water maze (MWM) analysis of acquisition performance of male (left) and female (right) mice represented as the mean distance (cm) that mice traveled to find the submerged platform plotted on the first trial of each training day (mean ± s.e.m.) Symbols represent statistically significant difference within the same genotype (*AD ASO-C vs. AD ASO-21 and #WT ASO-C vs. WT ASO-21) or between groups with same treatment ($^{\Delta}$AD ASO-C vs. WT ASO-C and ^AD ASO-21 vs. WT ASO-21) on the corresponding day (two-way repeated-measures ANOVA with Tukey multiple comparison test; $^{*}P < 0.05$, $^{\Delta}P < 0.05$, $^{\Delta\Delta}P < 0.01$).

 © 2016 The Authors

A

Gene	location	Intron length	Position (nucleotides)	mismatch	basepairs
METAP2	Intergenic	n/a	3051 upstream of AUG	C>G	16
SP4	Intron 3	36,682	13025 from 5' splice site	T>C	11
RUNX1	Intron 6	30,393	8188 from 5' splice site	C>T	9

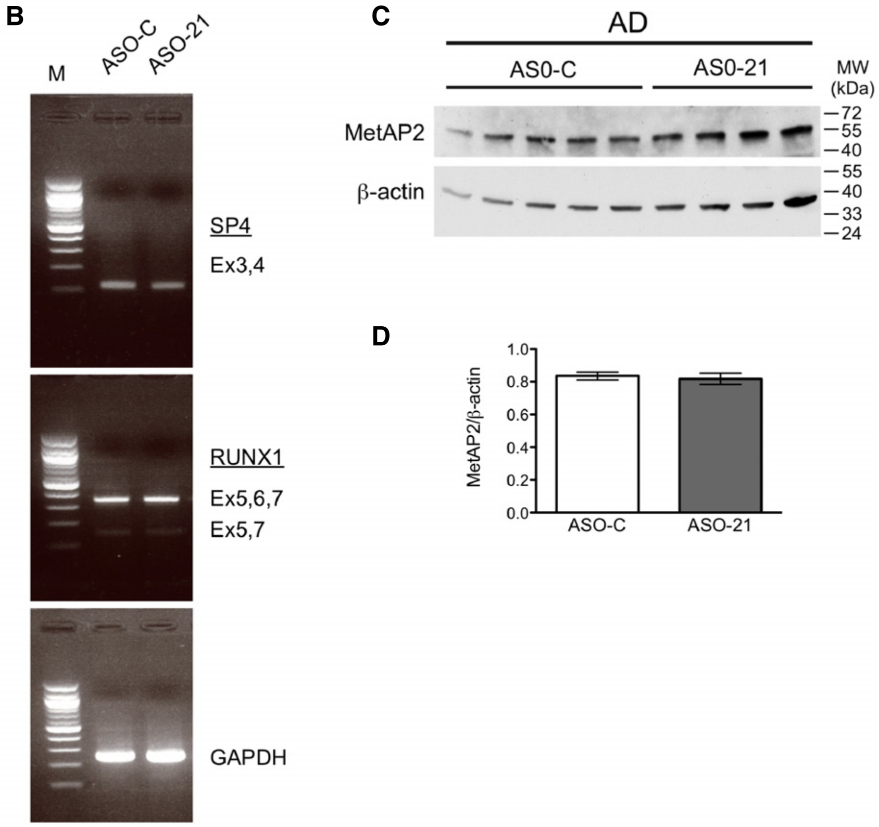

Figure EV6. ASO-21 does not affect splicing or expression of genomic targets with one mismatch.

A ASO-21 has a single mismatched nucleotide with three other sites in the mouse genome. The genes associated with these potential off-target binding sites are shown. The location of the site, intronic length, position relative to nearby landmarks, the specific mismatch, and contiguous base pairs in the putative duplex between ASO-21 and the off-target site are shown.

B RT–PCR analysis of RNA isolated from the cortex of AD mice treated with either ASO-C or ASO-21, as in Fig 4. The specific region of the transcript that is predicted to be affected by ASO-21 binding was amplified, and products were separated on a 2% agarose gel. No aberrant splicing or changes in alternative splicing patterns were observed.

C Immunoblot analysis of MetAP2 protein from the cortex of AD mice treated with either ASO-C or ASO-21, as in Fig 4. ASO-21 could form a putative duplex upstream of the MetAP2 start codon, potentially affecting expression.

D Graph depicting the quantitation of MetAP2 protein expression depicted in (C). Bars represent mean ± s.e.m.

Source data are available online for this figure.

© 2016 The Authors
