# Supplementary figures and images for: Therapeutic correction of ApoER2 splicing in Alzheimer's disease mice using antisense oligonucleotides
Source: EMBO Mol Med. 2016 Feb 22;8(4):328–45. doi: 10.15252/emmm.201505846 (PMC4818756; doi:10.15252/emmm.201505846)

Source Data: Expanded View Figure 1

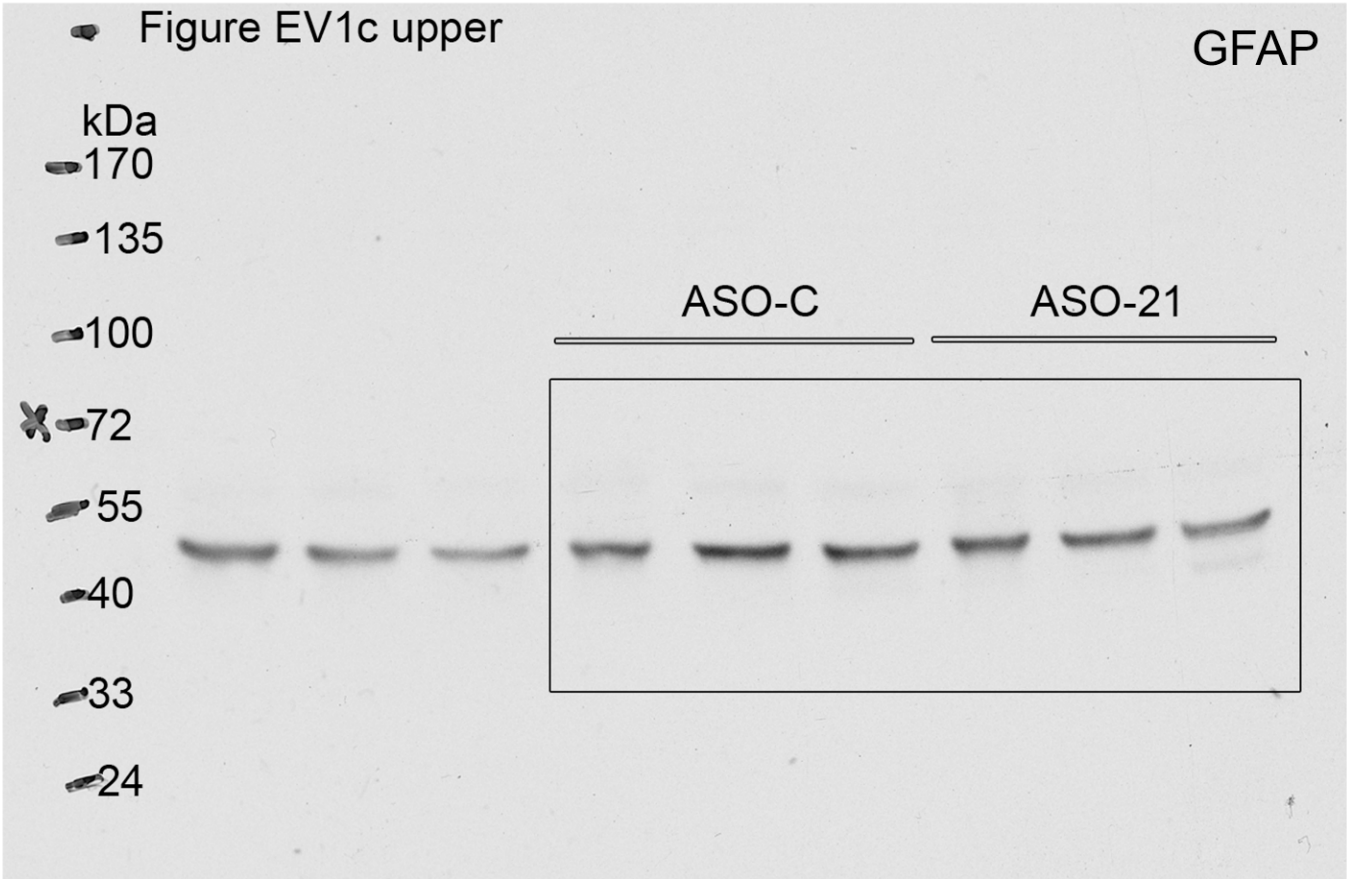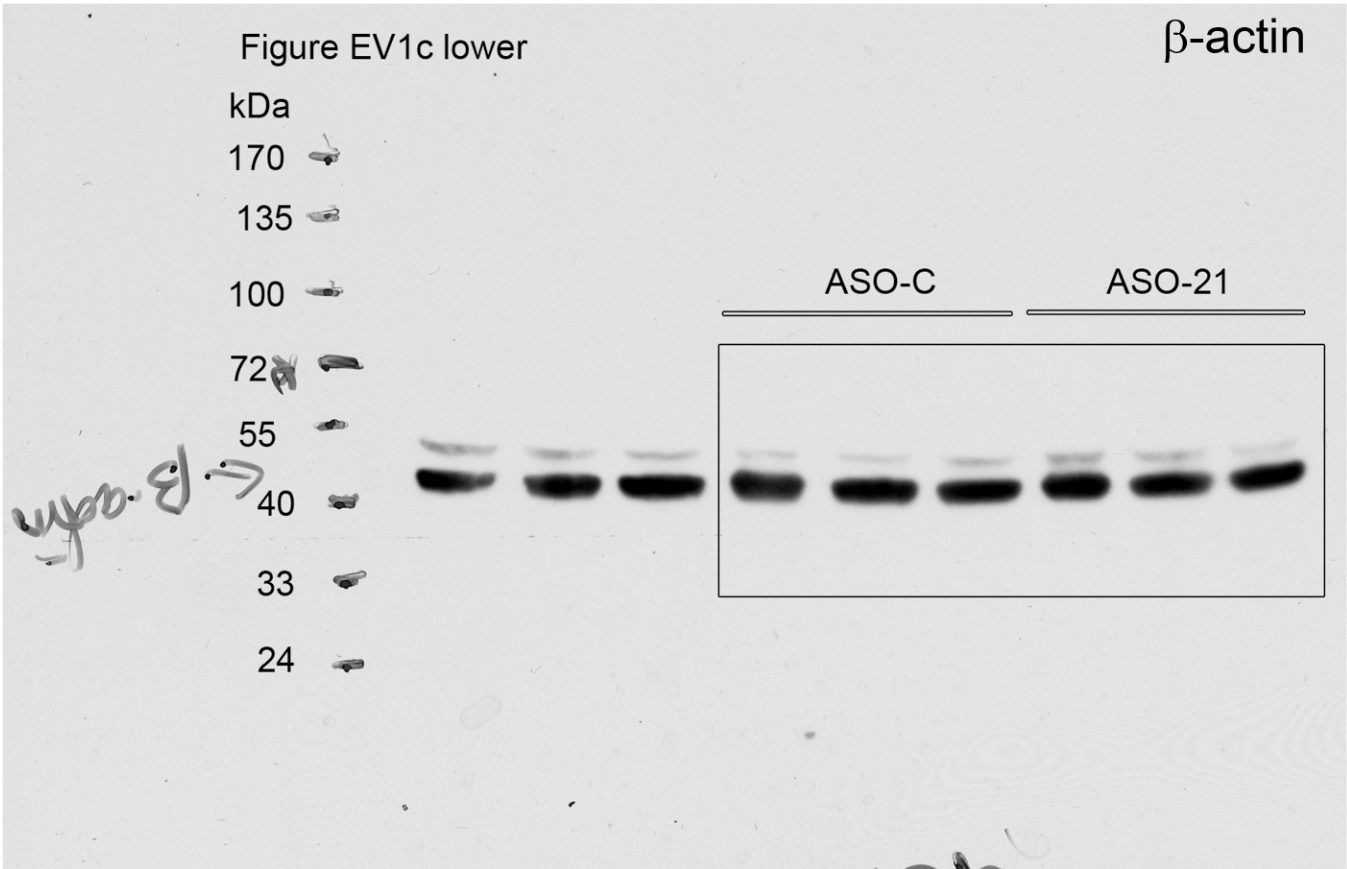

Supplement: Supplementary file 3 — Source Data for Expanded View [file EMMM-8-328-s008.zip › Source_Data_for_Expanded_View_and_Appendix/Source_data_for_EV_Figure_1.pdf]

# Source Data: Expanded View Figure 2

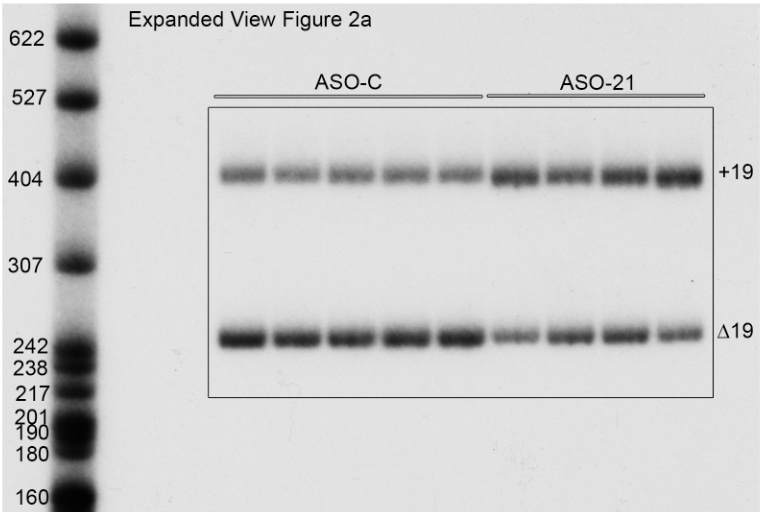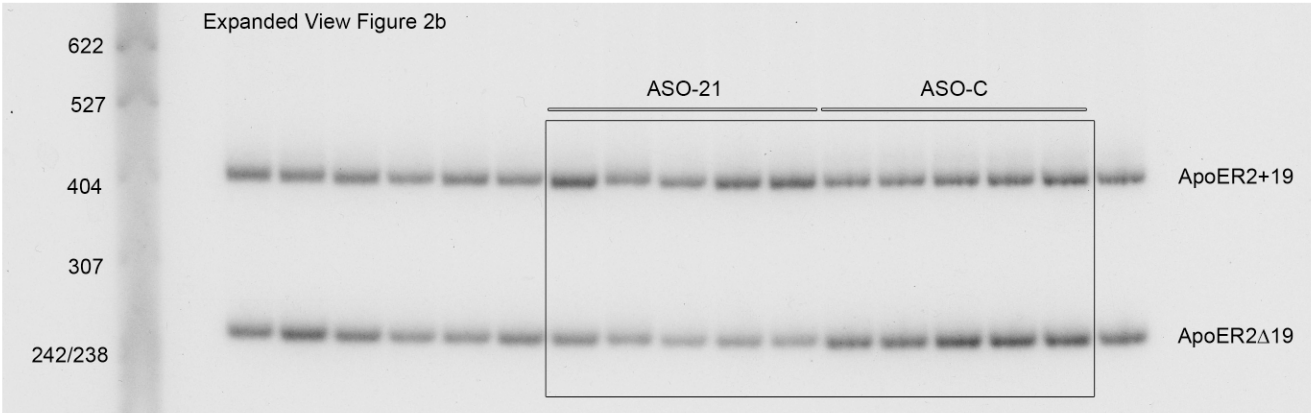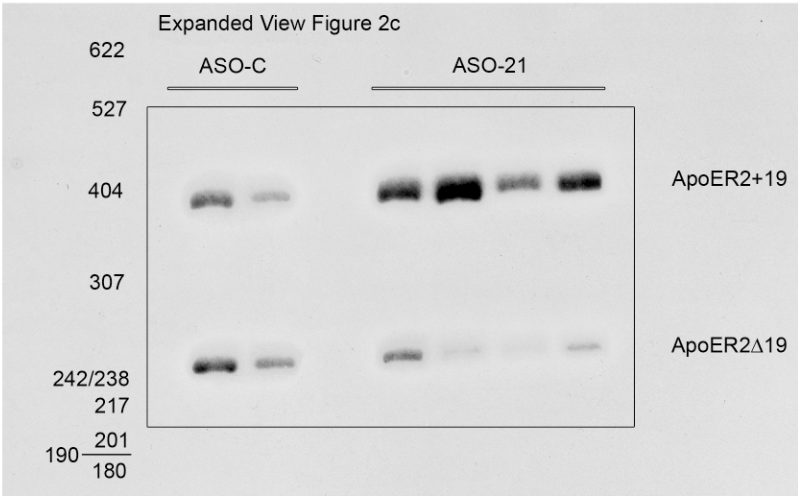

Supplement: Supplementary file 3 — Source Data for Expanded View [file EMMM-8-328-s008.zip › Source_Data_for_Expanded_View_and_Appendix/Source_data_for_EV_Figure_2.pdf]

Source Data: Expanded View Figure 6

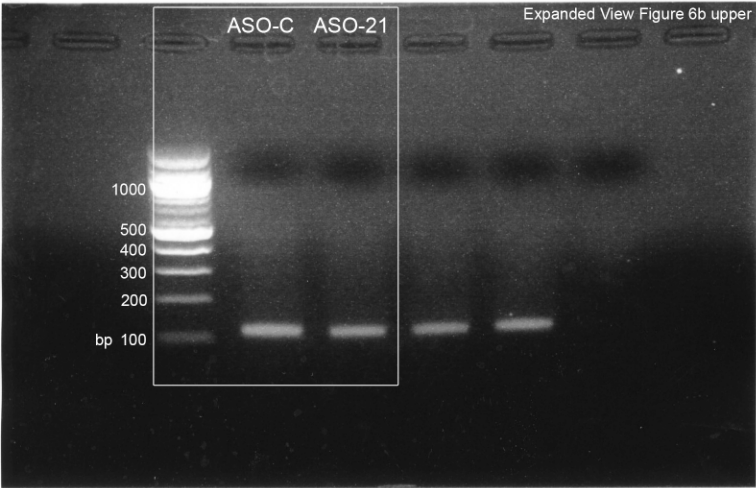

12-16-15 SP4

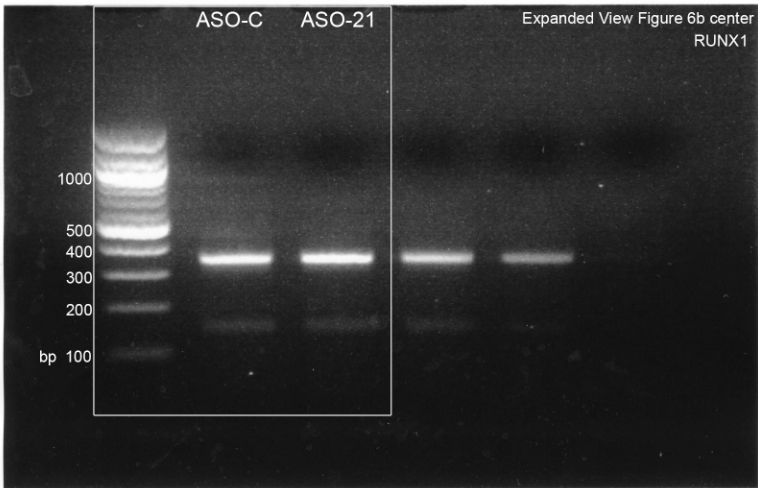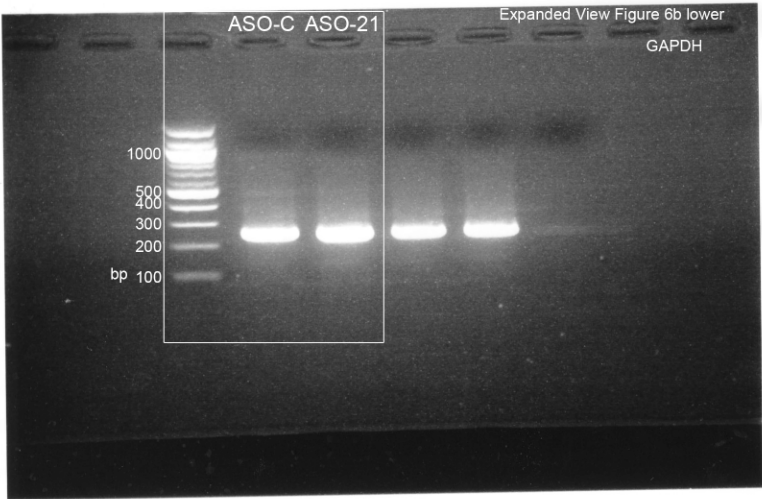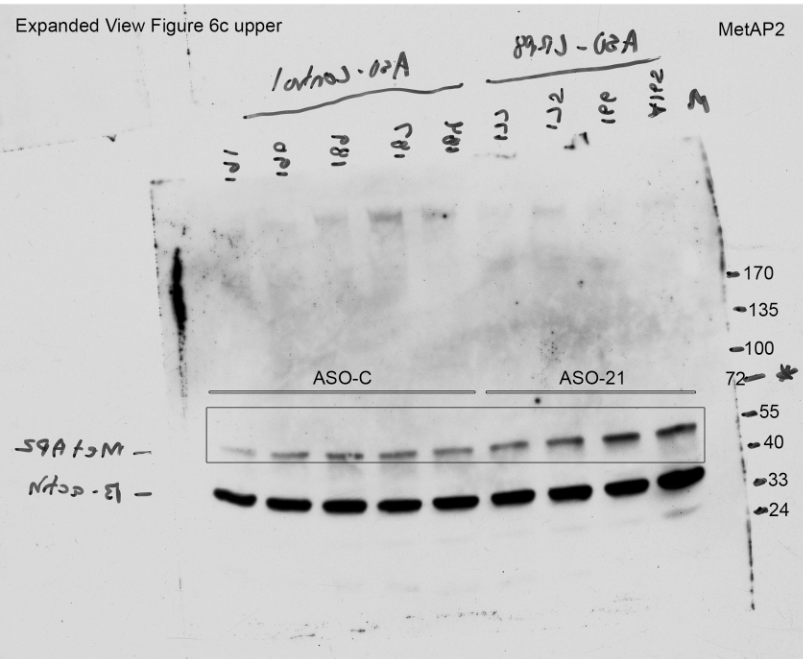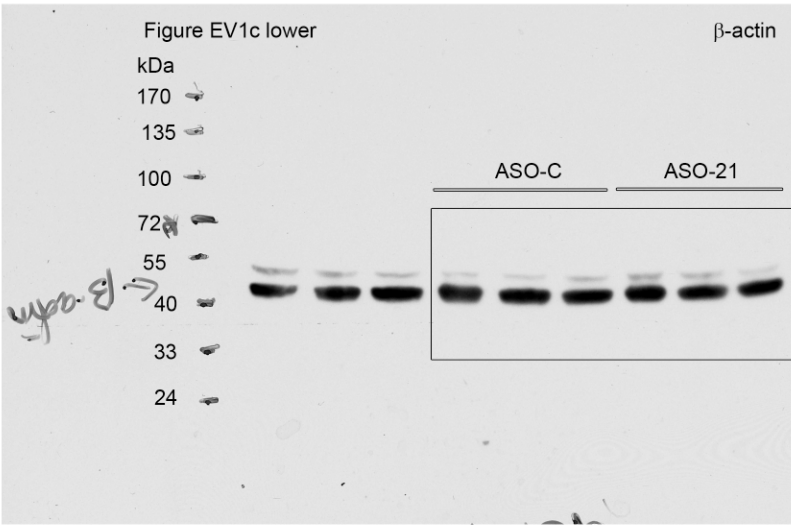

Supplement: Supplementary file 3 — Source Data for Expanded View [file EMMM-8-328-s008.zip › Source_Data_for_Expanded_View_and_Appendix/Source_data_for_EV_Figure_6.pdf]

Source Data: Figure 1

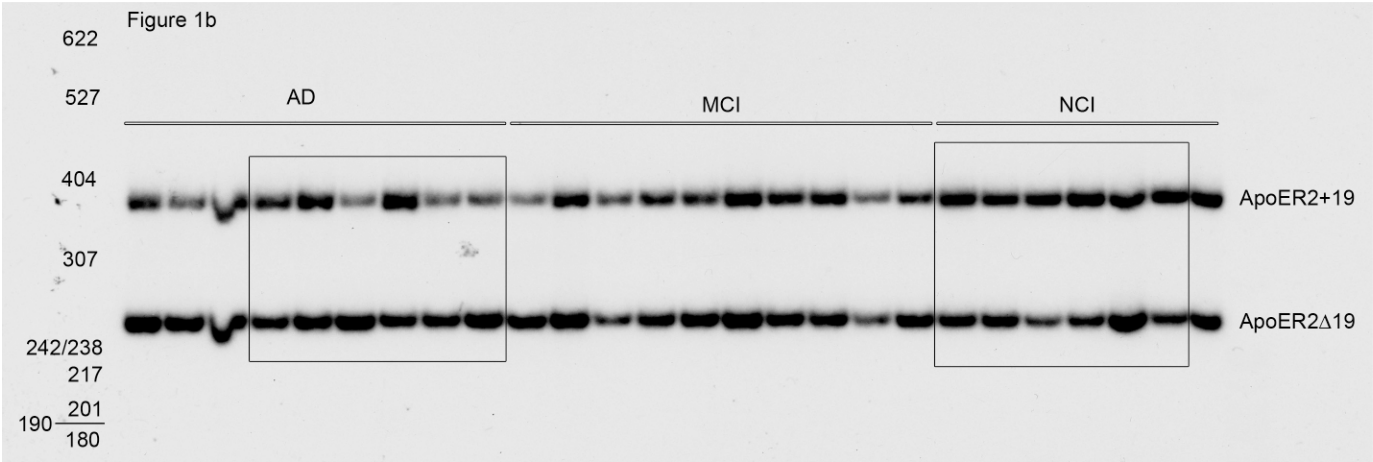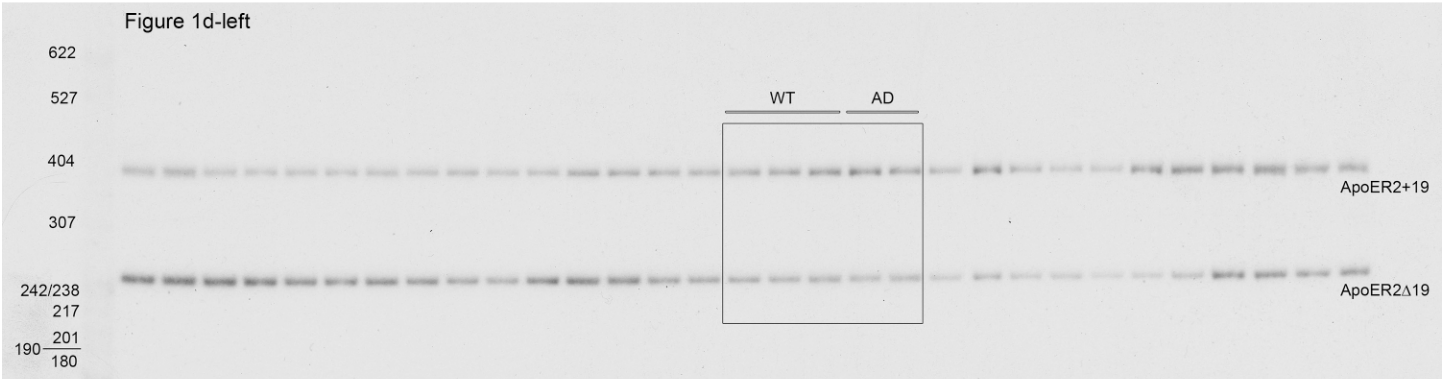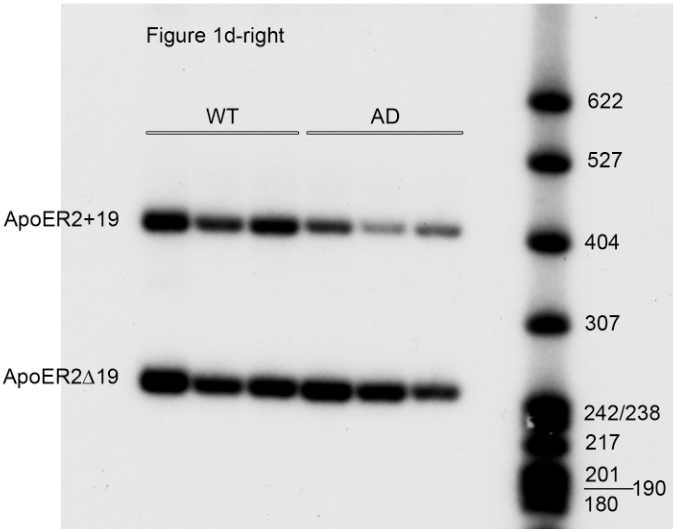

Supplement: Supplementary file 5 — Source Data for Figure 1 [file EMMM-8-328-s003.pdf]

Source data: Figure 2

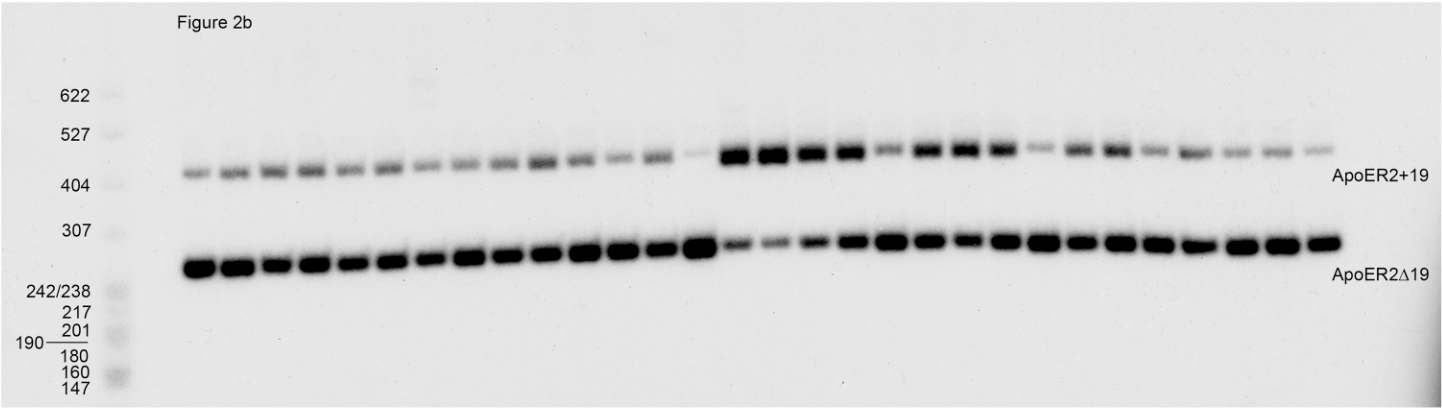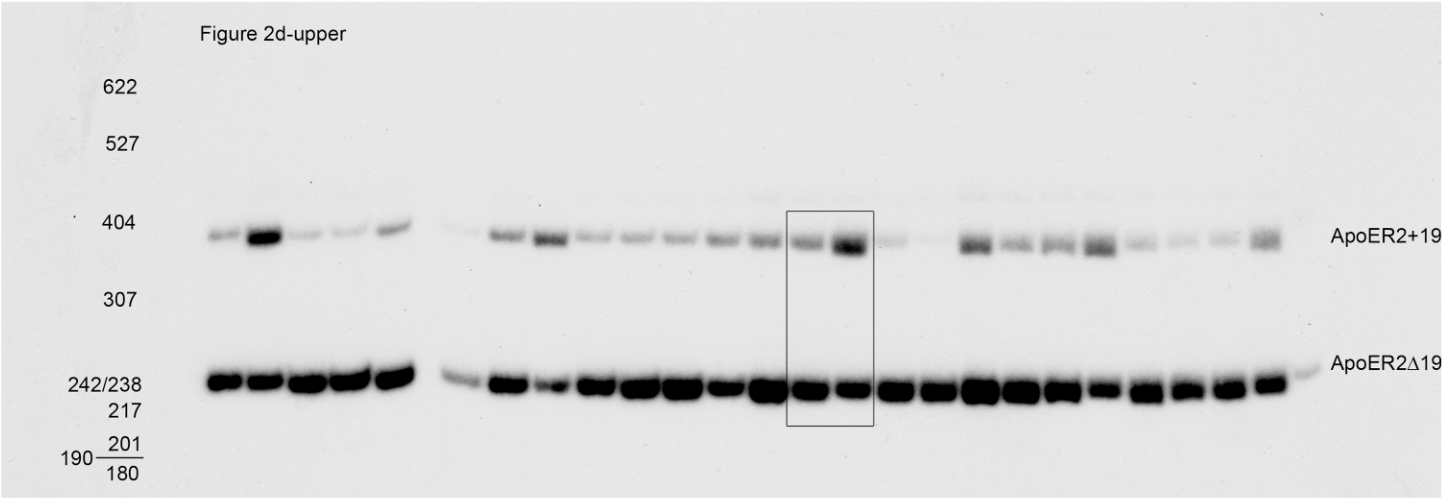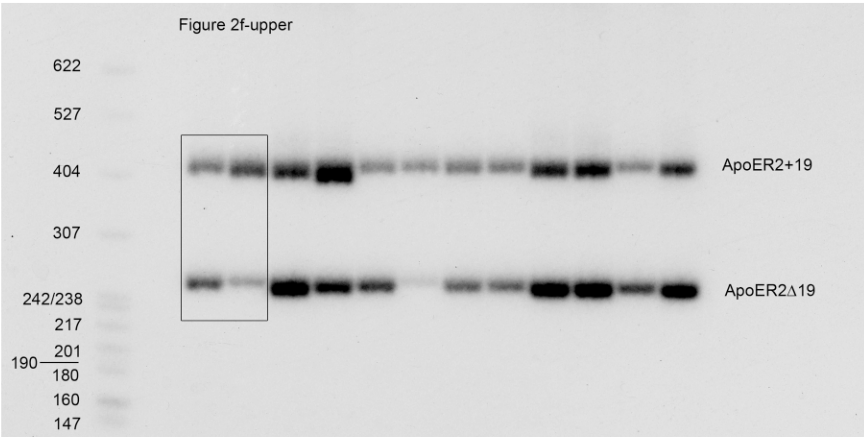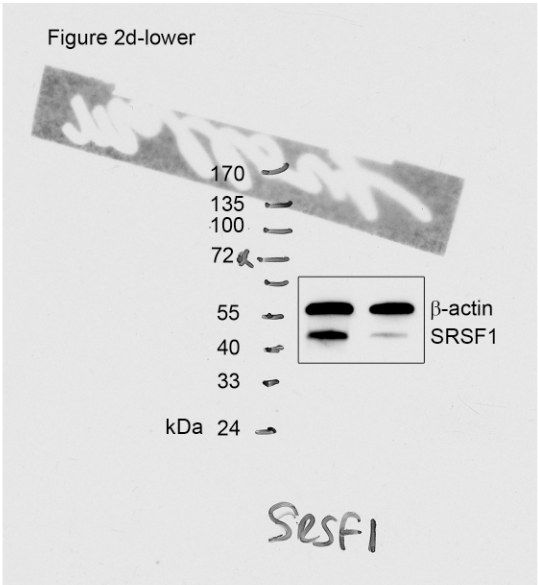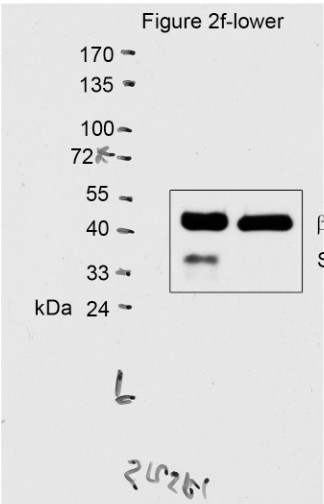

Supplement: Supplementary file 6 — Source Data for Figure 2 [file EMMM-8-328-s004.pdf]

Source data: Figure 3

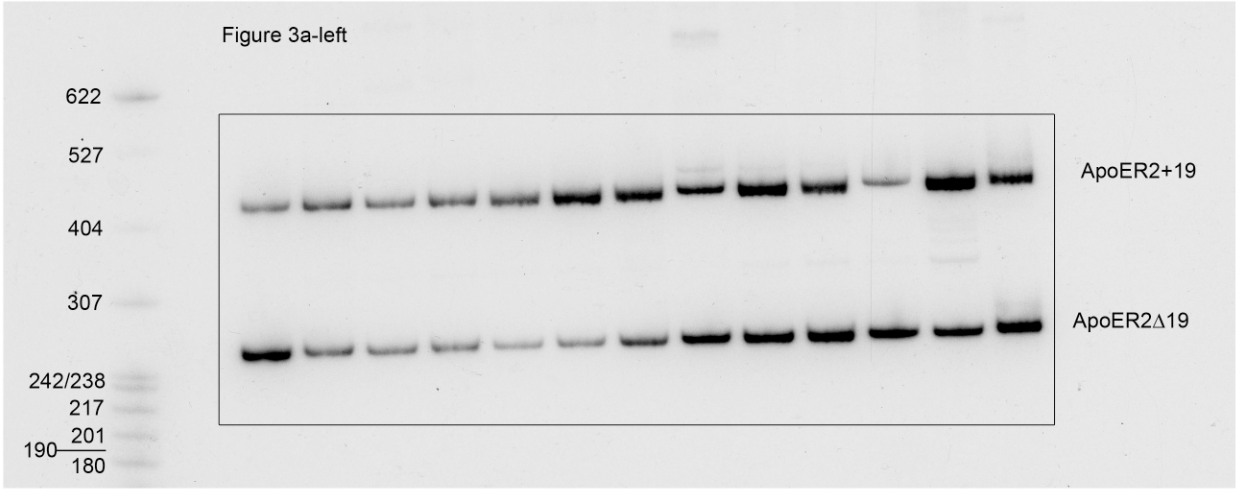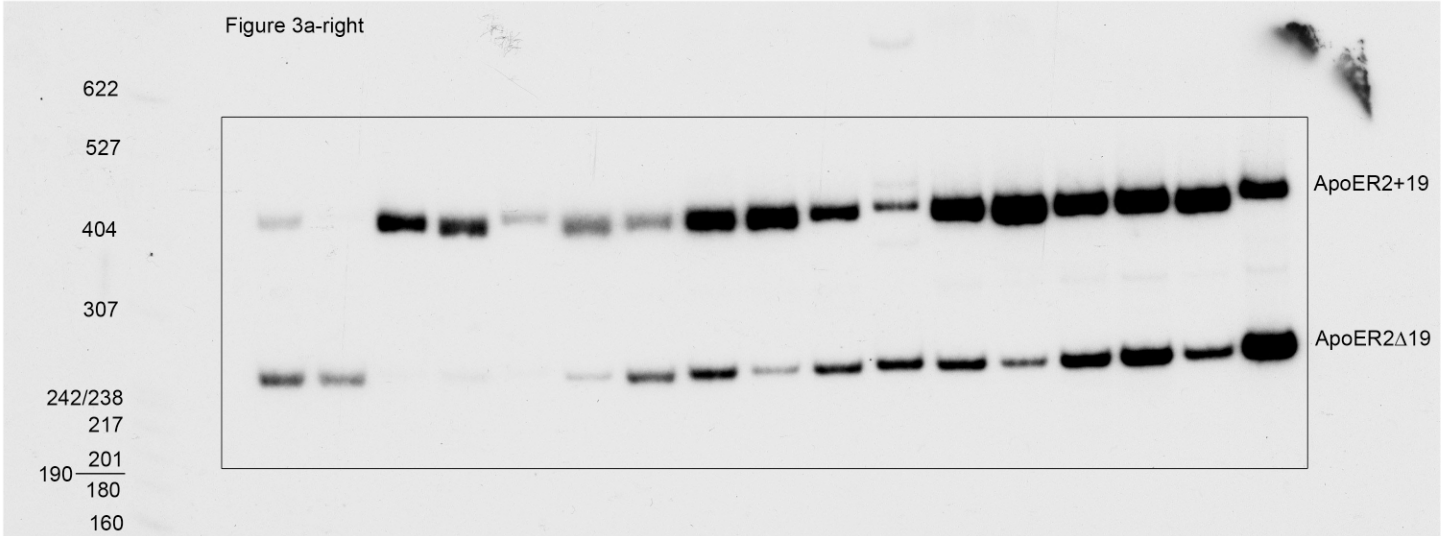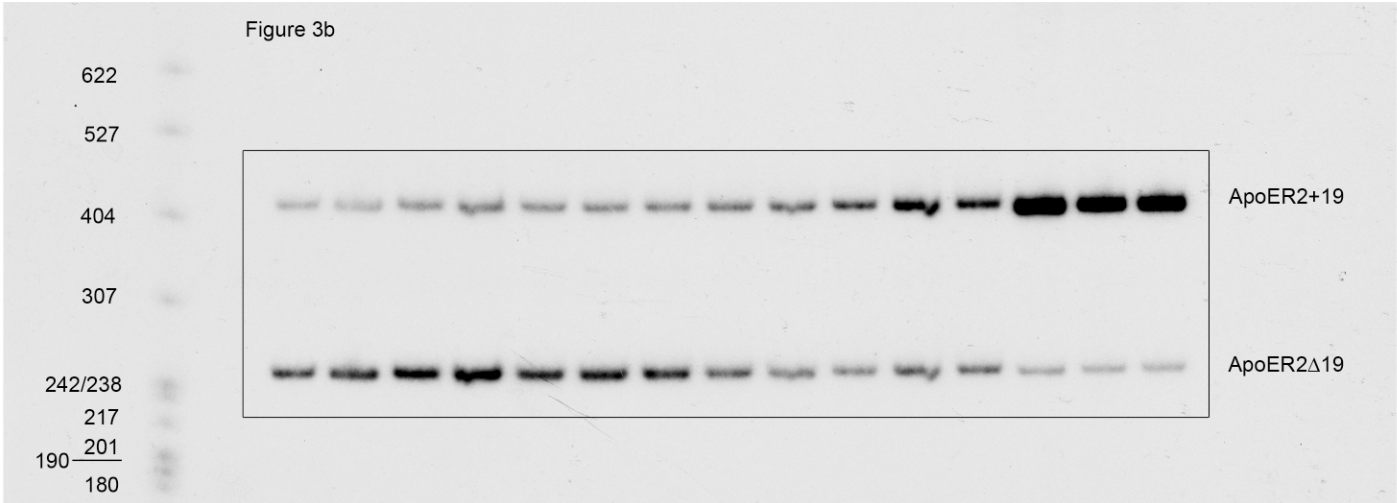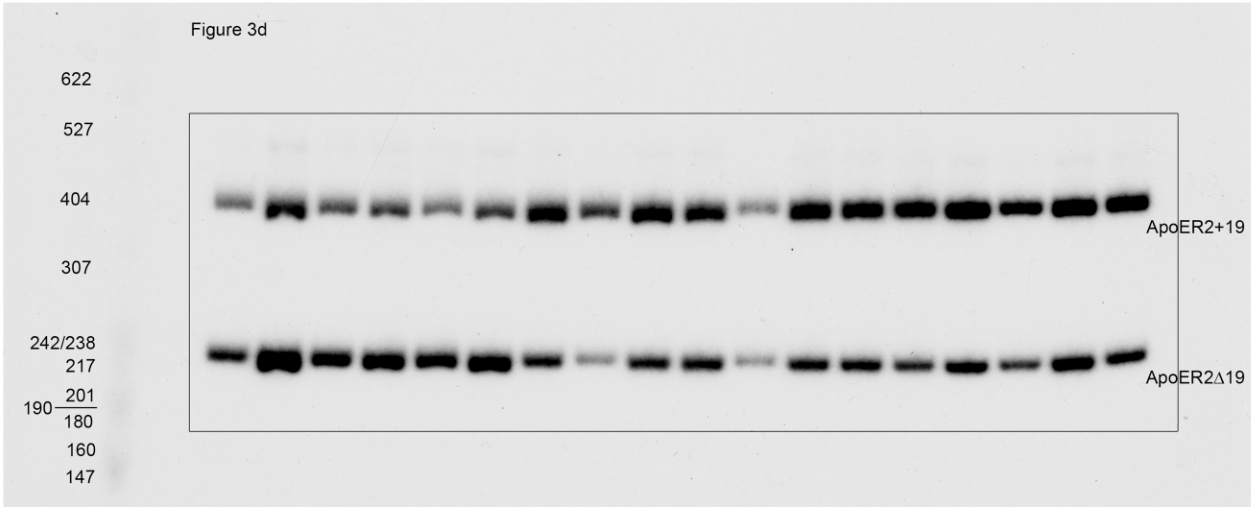

Supplement: Supplementary file 7 — Source Data for Figure 3 [file EMMM-8-328-s005.pdf]

Source data: Figure 4

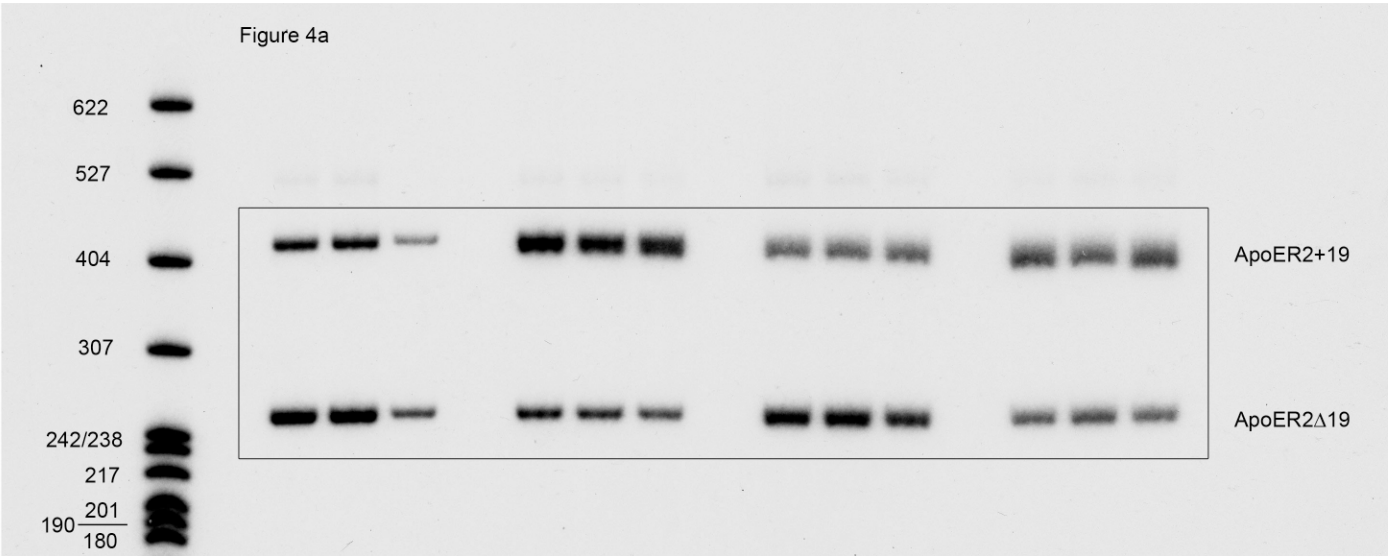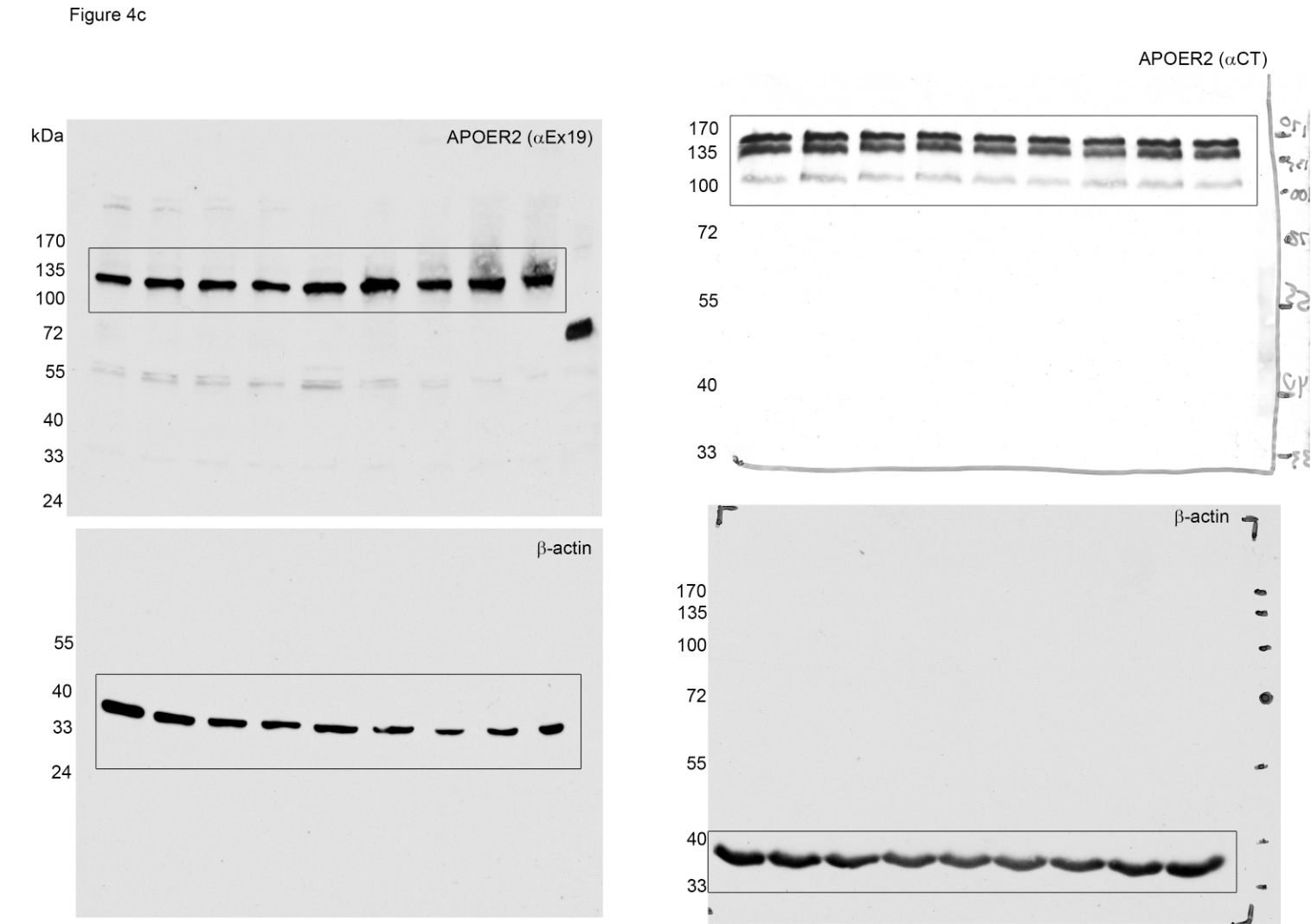

Supplement: Supplementary file 8 — Source Data for Figure 4 [file EMMM-8-328-s006.pdf]
