# Supplementary material for: Therapeutic correction of ApoER2 splicing in Alzheimer's disease mice using antisense oligonucleotides
Source: EMBO Mol Med. 2016 Feb 22;8(4):328–45. doi: 10.15252/emmm.201505846 (PMC4818756; doi:10.15252/emmm.201505846)
Supplement: Supplementary file 9 — Source Data for Figure 6 [file EMMM-8-328-s007.docx]

| **FIGURE 6B:** MALES |  |  |  |  |  |
| --- | --- | --- | --- | --- | --- |
| Two-way RM ANOVA | Matching: Stacked |  |  |  |  |
| Alpha | 0.05 |  |  |  |  |
|  |  |  |  |  |  |
| **Source of Variation** | **% of total variation** | **P value** | **P value summary** | **Significant?** |  |
| Interaction | 4.681 | 0.0053 | ** | Yes |  |
| Time (Day) | 17.01 | < 0.0001 | **** | Yes |  |
| Genotype/Treatment | 32.68 | < 0.0001 | **** | Yes |  |
| Subjects (matching) | 20.88 | < 0.0001 | **** | Yes |  |
|  |  |  |  |  |  |
| **ANOVA table** | **SS** | **DF** | **MS** | **F (DFn, DFd)** | **P value** |
| Interaction | 896349 | 9 | 99594 | F (9, 132) = 2.773 | P = 0.0053 |
| Time (Day) | 3.257e+006 | 3 | 1.086e+006 | F (3, 132) = 30.23 | P < 0.0001 |
| Genotype/Treatment | 6.257e+006 | 3 | 2.086e+006 | F (3, 44) = 22.96 | P < 0.0001 |
| Subjects (matching) | 3.997e+006 | 44 | 90848 | F (44, 132) = 2.530 | P < 0.0001 |
| Residual | 4.740e+006 | 132 | 35910 |  |  |
|  |  |  |  |  |  |
|  |  |  |  |  |  |
| Number of families | 4 |  |  |  |  |
| Number of comparisons per family | 6 |  |  |  |  |
| **Alpha** | **0.05** |  |  |  |  |
|  |  |  |  |  |  |
| **Tukey's multiple comparisons test** | **Mean Diff.** | **95% CI of diff.** | **Significant?** | **Summary** | **Adjusted P Value** |
|  |  |  |  |  |  |
| DAY 1 |  |  |  |  |  |
| AD ASO-C (n=12) vs. AD ASO-21 (n=12) | 55.85 | -180.1 to 291.8 | No | ns | 0.9275 |
| AD ASO-C (n=12) vs. WT ASO-C (n=12) | 166.2 | -69.73 to 402.1 | No | ns | 0.2641 |
| AD ASO-C (n=12) vs. WT ASO-21 (n=12) | 209.4 | -26.57 to 445.3 | No | ns | 0.1015 |
| AD ASO-21 (n=12) vs. WT ASO-C (n=12) | 110.3 | -125.6 to 346.3 | No | ns | 0.6194 |
| AD ASO-21 (n=12) vs. WT ASO-21 (n=12) | 153.5 | -82.42 to 389.4 | No | ns | 0.3333 |
| WT ASO-C (n=12) vs. WT ASO-21 (n=12) | 43.16 | -192.8 to 279.1 | No | ns | 0.9646 |
|  |  |  |  |  |  |
| DAY 2 |  |  |  |  |  |
| AD ASO-C (n=12) vs. AD ASO-21 (n=12) | 310.2 | 74.24 to 546.1 | Yes | ** | 0.0044 |
| AD ASO-C (n=12) vs. WT ASO-C (n=12) | 641.7 | 405.7 to 877.6 | Yes | ΔΔΔΔ | < 0.0001 |
| AD ASO-C (n=12) vs. WT ASO-21 (n=12) | 571.5 | 335.6 to 807.4 | Yes | 🞆🞆🞆🞆 | < 0.0001 |
| AD ASO-21 (n=12) vs. WT ASO-C (n=12) | 331.5 | 95.58 to 567.4 | Yes | •• | 0.0020 |
| AD ASO-21 (n=12) vs. WT ASO-21 (n=12) | 261.3 | 25.38 to 497.2 | Yes | ^ | 0.0234 |
| WT ASO-C (n=12) vs. WT ASO-21 (n=12) | -70.19 | -306.1 to 165.7 | No | ns | 0.8671 |
|  |  |  |  |  |  |
| DAY 3 |  |  |  |  |  |
| AD ASO-C (n=12) vs. AD ASO-21 (n=12) | 304.8 | 68.84 to 540.7 | Yes | ** | 0.0054 |
| AD ASO-C (n=12) vs. WT ASO-C (n=12) | 515.4 | 279.4 to 751.3 | Yes | ΔΔΔΔ | < 0.0001 |
| AD ASO-C (n=12) vs. WT ASO-21 (n=12) | 462.4 | 226.5 to 698.4 | Yes | 🞆🞆🞆🞆 | < 0.0001 |
| AD ASO-21 (n=12) vs. WT ASO-C (n=12) | 210.6 | -25.34 to 446.5 | No | ns | 0.0985 |
| AD ASO-21 (n=12) vs. WT ASO-21 (n=12) | 157.7 | -78.27 to 393.6 | No | ns | 0.3096 |
| WT ASO-C (n=12) vs. WT ASO-21 (n=12) | -52.93 | -288.9 to 183.0 | No | ns | 0.9374 |
|  |  |  |  |  |  |
| DAY 4 |  |  |  |  |  |
| AD ASO-C (n=12) vs. AD ASO-21 (n=12) | 131.9 | -104.0 to 367.9 | No | ns | 0.4698 |
| AD ASO-C (n=12) vs. WT ASO-C (n=12) | 479.1 | 243.1 to 715.0 | Yes | ΔΔΔΔ | < 0.0001 |
| AD ASO-C (n=12) vs. WT ASO-21 (n=12) | 403.9 | 168.0 to 639.9 | Yes | 🞆🞆🞆🞆 | < 0.0001 |
| AD ASO-21 (n=12) vs. WT ASO-C (n=12) | 347.1 | 111.2 to 583.1 | Yes | •• | 0.0011 |
| AD ASO-21 (n=12) vs. WT ASO-21 (n=12) | 272.0 | 36.06 to 507.9 | Yes | ^ | 0.0167 |
| WT ASO-C (n=12) vs. WT ASO-21 (n=12) | -75.13 | -311.1 to 160.8 | No | ns | 0.8421 |

| **FIGURE 6B:** FEMALES |  |  |  |  |  | |
| --- | --- | --- | --- | --- | --- | --- |
| Two-way RM ANOVA | Matching: Stacked |  |  |  |  | |
| **Alpha** | **0.05** |  |  |  |  | |
|  |  |  |  |  |  | |
| **Source of Variation** | **% of total variation** | **P value** | **P value summary** | **Significant?** |  | |
| Interaction | 3.295 | 0.0438 | * | Yes |  | |
| Time (Day) | 17.08 | < 0.0001 | **** | Yes |  | |
| Genotype/Treatment | 15.44 | 0.0005 | *** | Yes |  | |
| Subjects (matching) | 36.59 | < 0.0001 | **** | Yes |  | |
|  |  |  |  |  | |  |
| **ANOVA table** | **SS** | **DF** | **MS** | **F (DFn, DFd)** | | **P value** |
| Interaction | 608917 | 9 | 67657 | F (9, 150) = 1.993 | P =0.0438 | |
| Time (Day) | 3.157e+006 | 3 | 1.052e+006 | F (3, 150) = 30.99 | P < 0.0001 | |
| Genotype/Treatment | 2.852e+006 | 3 | 950816 | F (3, 50) = 7.032 | P = 0.0005 | |
| Subjects (matching) | 6.761e+006 | 50 | 135214 | F (50, 150) = 3.983 | P < 0.0001 | |
| Residual | 5.093e+006 | 150 | 33951 |  |  | |
|  |  |  |  |  |  | |
|  |  |  |  |  |  | |
| Number of families | 4 |  |  |  |  | |
| Number of comparisons per family | 6 |  |  |  |  | |
| **Alpha** | **0.05** |  |  |  |  | |
|  |  |  |  |  |  | |
| **Tukey's multiple comparisons test** | **Mean Diff.** | **95% CI of diff.** | **Significant?** | **Summary** | **Adjusted P Value** | |
|  |  |  |  |  |  | |
| **DAY 1** |  |  |  |  |  | |
| AD ASO-C (n=15) vs. AD ASO-LRP8 (n=11) | -75.73 | -326.1 to 174.6 | No | ns | 0.8618 | |
| AD ASO-C (n=15) vs. WT ASO-C (n=13) | 58.59 | -180.4 to 297.6 | No | ns | 0.9206 | |
| AD ASO-C (n=15) vs. WT ASO-LRP8 (n=15) | 57.34 | -173.0 to 287.6 | No | ns | 0.9172 | |
| AD ASO-LRP8 (n=11) vs. WT ASO-C (n=13) | 134.3 | -124.1 to 392.7 | No | ns | 0.5343 | |
| AD ASO-LRP8 (n=11) vs. WT ASO-LRP8 (n=15) | 133.1 | -117.3 to 383.4 | No | ns | 0.5153 | |
| WT ASO-C (n=13) vs. WT ASO-LRP8 (n=15) | -1.252 | -240.3 to 237.7 | No | ns | > 0.9999 | |
|  |  |  |  |  |  | |
| **DAY 2** |  |  |  |  |  | |
| AD ASO-C (n=15) vs. AD ASO-LRP8 (n=11) | -9.970 | -260.3 to 240.4 | No | ns | 0.9996 | |
| AD ASO-C (n=15) vs. WT ASO-C (n=13) | 284.0 | 45.00 to 523.0 | Yes | Δ | 0.0126 | |
| AD ASO-C (n=15) vs. WT ASO-LRP8 (n=15) | 283.5 | 53.15 to 513.8 | Yes | 🞆🞆 | 0.0089 | |
| AD ASO-LRP8 (n=11) vs. WT ASO-C (n=13) | 294.0 | 35.58 to 552.4 | Yes | • | 0.0187 | |
| AD ASO-LRP8 (n=11) vs. WT ASO-LRP8 (n=15) | 293.4 | 43.06 to 543.8 | Yes | ^ | 0.0143 | |
| WT ASO-C (n=13) vs. WT ASO-LRP8 (n=15) | -0.5407 | -239.5 to 238.5 | No | ns | > 0.9999 | |
|  |  |  |  |  |  | |
| **DAY 3** |  |  |  |  |  | |
| AD ASO-C (n=15) vs. AD ASO-LRP8 (n=11) | 161.7 | -88.63 to 412.1 | No | ns | 0.3403 | |
| AD ASO-C (n=15) vs. WT ASO-C (n=13) | 375.4 | 136.4 to 614.4 | Yes | ΔΔΔ | 0.0004 | |
| AD ASO-C (n=15) vs. WT ASO-LRP8 (n=15) | 292.3 | 62.00 to 522.6 | Yes | 🞆🞆 | 0.0065 | |
| AD ASO-LRP8 (n=11) vs. WT ASO-C (n=13) | 213.7 | -44.71 to 472.1 | No | ns | 0.1434 | |
| AD ASO-LRP8 (n=11) vs. WT ASO-LRP8 (n=15) | 130.6 | -119.8 to 380.9 | No | ns | 0.5316 | |
| WT ASO-C (n=13) vs. WT ASO-LRP8 (n=15) | -83.11 | -322.1 to 155.9 | No | ns | 0.8043 | |
|  |  |  |  |  |  | |
| **DAY 4** |  |  |  |  |  | |
| AD ASO-C (n=15) vs. AD ASO-LRP8 (n=11) | -35.69 | -286.1 to 214.7 | No | ns | 0.9828 | |
| AD ASO-C (n=15) vs. WT ASO-C (n=13) | 250.5 | 11.54 to 489.5 | Yes | Δ | 0.0359 | |
| AD ASO-C (n=15) vs. WT ASO-LRP8 (n=15) | 273.9 | 43.56 to 504.2 | Yes | 🞆 | 0.0125 | |
| AD ASO-LRP8 (n=11) vs. WT ASO-C (n=13) | 286.2 | 27.84 to 544.6 | Yes | • | 0.0234 | |
| AD ASO-LRP8 (n=11) vs. WT ASO-LRP8 (n=15) | 309.6 | 59.19 to 559.9 | Yes | ΛΛ | 0.0085 | |
| WT ASO-C (n=13) vs. WT ASO-LRP8 (n=15) | 23.32 | -215.7 to 262.3 | No | ns | 0.9943 | |
